# A Moldy Application of MALDI: MALDI-ToF Mass Spectrometry for Fungal Identification

**DOI:** 10.3390/jof5010004

**Published:** 2019-01-03

**Authors:** Robin Patel

**Affiliations:** 1 Department of Laboratory Medicine and Pathology, Division of Clinical Microbiology, Mayo Clinic, Rochester, MN 55905, USA; patel.robin@mayo.edu; Tel.: +1-507-538-0579; Fax: +1-507-284-4272; 2 Department of Medicine, Mayo Clinic, Division of Infectious Diseases, Rochester, MN 55905, USA

**Keywords:** MALDI-ToF MS, yeast, fungus

## Abstract

As a result of its being inexpensive, easy to perform, fast and accurate, matrix-assisted laser desorption ionization time-of-flight mass spectrometry (MALDI-ToF MS) is quickly becoming the standard means of bacterial identification from cultures in clinical microbiology laboratories. Its adoption for routine identification of yeasts and even dimorphic and filamentous fungi in cultures, while slower, is now being realized, with many of the same benefits as have been recognized on the bacterial side. In this review, the use of MALDI-ToF MS for identification of yeasts, and dimorphic and filamentous fungi grown in culture will be reviewed, with strengths and limitations addressed.

## 1. Background and Introduction

The concept of using mass spectrometry for bacterial identification was suggested by Catherine Fenselau and John Anhalt in 1975 [1], but at the time, intact proteins were not analyzable due to fragmentation during the mass spectrometry (MS) process, with mass spectrometric analysis of intact proteins only becoming possible a decade later. In 1985, Koichi Tanaka described a “soft desorption ionization” technique allowing mass spectrometry of biological macromolecules achieved using ultrafine metal powder and glycerol; for his discovery, he was awarded the Nobel Prize in Chemistry [2]. About the same time, Franz Hillenkamp and Michael Karas reported a soft desorption ionization using an organic compound matrix [3]; it was their approach for which the designation “matrix-assisted laser desorption ionization” or MALDI, was coined and on which subsequent clinical microbiology applications were based. Tied with time-of-flight or ToF analysis, this advancement made it possible to perform mass spectrometry on intact bacterial and, ultimately, fungal cells. However, it took until advances in informatics allowed the connection of microbial MALDI-ToF MS databases to automated computer-based analytics for MALDI-ToF MS to ultimately become usable for routine identification of bacteria and, eventually, fungi in clinical laboratories. In due course, these developments led to the commercialization and, ultimately, regulatory approval of MALDI-ToF MS systems for clinical microbiology laboratories. Although initial applications of MALDI-ToF MS for rapid, inexpensive identification of microorganisms in culture focused on bacteria, it was quickly realized that this new technology could be equally applied to yeasts and, with some caveats, dimorphic and filamentous fungi.

**Figure 1:**
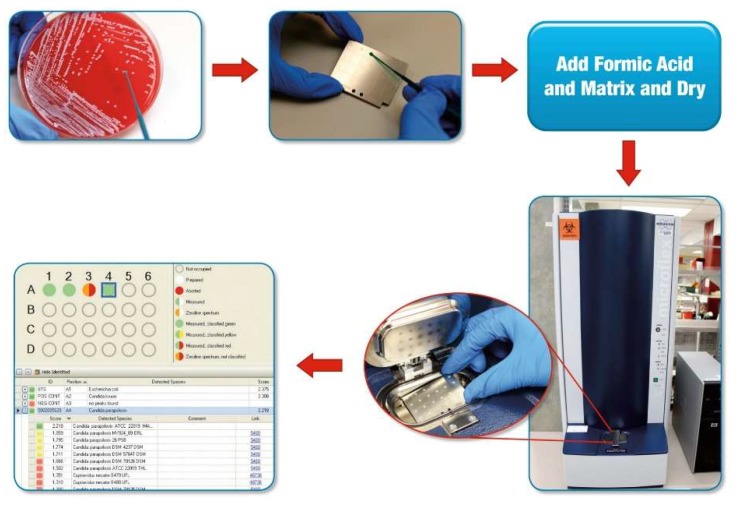
Process of matrix-assisted laser desorption ionization time-of-flight mass spectrometry (MALDI-ToF MS) for yeast identification [7,8]. A colony is picked from a culture plate to a spot on a MALDI-ToF MS target plate (a disposable or reusable plate with a number of spots, each of which may be used to test different colonies). For yeast applications, cells are typically treated with formic acid on the target plate, followed by drying. The spot is overlain with 1–2 μL of matrix and dried. The plate is placed in the ionization chamber of the mass spectrometer (Figure 2). A mass spectrum is produced and compared against a library of mass spectra by the software, resulting in identification of the yeast (*Candida parapsilosis* in position A4 in the example). Used with permission of the Mayo Foundation for Medical Education and Research. All rights reserved.

**Figure 2:**
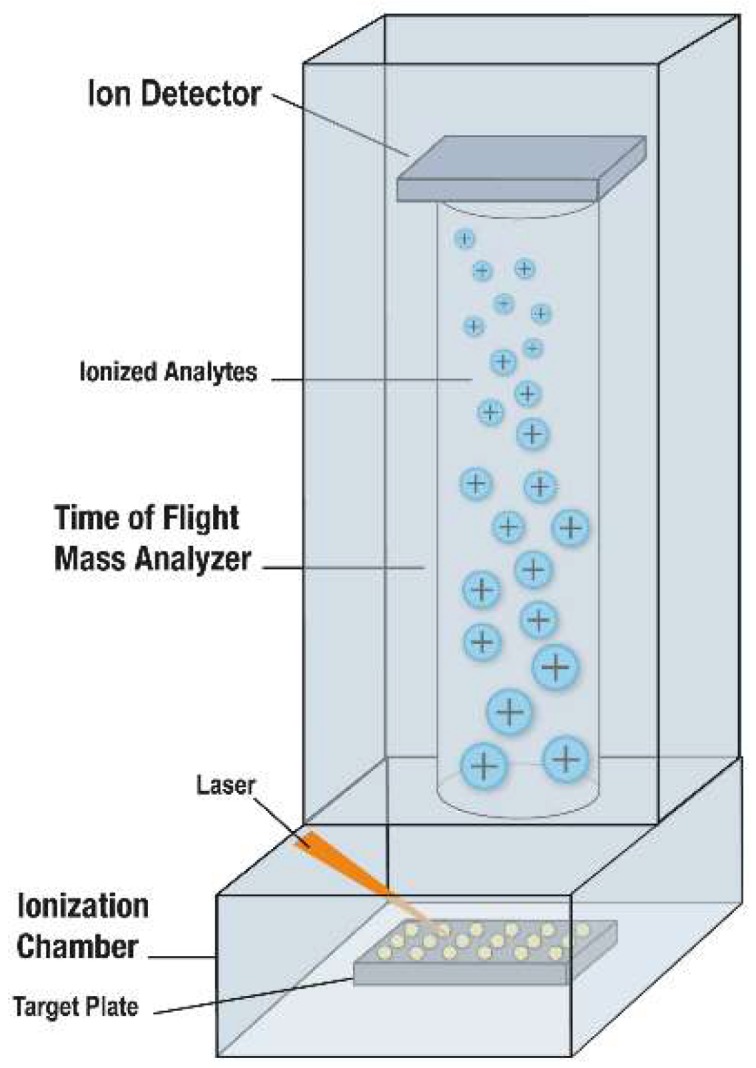
Mass spectrometer used for MALDI-ToF MS [7,8]. The MALDI-ToF MS plate is placed into the chamber of the instrument. Each spot to be analyzed is shot by a laser, resulting in desorption and ionization of bacterial or fungal and matrix molecules from the target plate. The cloud of ionized molecules is accelerated into the time-of-flight mass analyzer, toward a detector. Lighter molecules travel quicker, followed by progressively heavier ones. A mass spectrum is produced; it denotes the number of ions hitting the detector with time. Separation is by mass-to-charge ratio; because charge is typically single for this application, separation is by molecular weight. Used with permission of the Mayo Foundation for Medical Education and Research. All rights reserved.

MALDI designates matrix that assists in desorption and ionization of highly abundant bacterial or fungal proteins through laser energy [4]. Like bacteria, fungi may be tested either by “direct transfer” to a MALDI-ToF MS target plate, with or without the addition of an on-plate formic acid treatment (to lyse cells, also referred to as “on-plate extraction” and “extended direct transfer”), or following a more formal (and time-consuming) off-plate protein extraction step. The former is most commonly used for yeasts and the latter for filamentous fungi. For direct transfer testing, whole cells from colonies are simply moved to the target plate using a loop, plastic or wooden stick, or pipette tip, to a “spot” on a MALDI-ToF MS target plate (a reusable or disposable plate with multiple test spots) (Figure 1). For on-plate formic acid treatment, a formic acid solution is incorporated, either by adding the formic acid solution prior to colony transfer or by overlaying the transferred colony with formic acid solution, followed by drying. Then, the microbial mass, either alone or after formic acid treatment, is overlain with matrix; following drying of the matrix, the target plate is moved into a mass spectrometer (Figure 2). After this point, the rest is automated vis-à-vis the described clinical microbiology applications. The matrix (e.g., α-cyano-4-hydroxycinnamic acid dissolved in 50% acetonitrile and 2.5% trifluoroacetic acid), which is used for bacteria and fungi alike, isolates microbial molecules from one another, protecting them from breaking up and allowing their desorption by laser energy; a majority of the energy is absorbed by the matrix, changing it to an ionized state. As a result of random impacts occurring in the gas phase, charge is moved from matrix to microbial molecules; ionized microbial molecules are then accelerated through a positively charged electrostatic field into a ToF, tube, which is under vacuum. In the tube, ions travel to an ion detector, with smaller analytes migrating fastest, followed by increasingly larger analytes; a mass spectrum is thereby generated, signifying the quantity of ions of a specified mass hitting the detector over time. The resultant mass spectrum represents the most abundant proteins, mainly ribosomal proteins, though with this application the specific proteins generating the mass spectrum are not separately identified. The overall mass spectrum is used as a signature profile of individual fungi (or bacteria), with peaks specific to groups, complexes, genera and/or species, depending on relatedness of the test organisms to other closely related ones. The mass spectrum of an individual isolate is compared to a database or library of reference spectra, producing a list of the most closely interrelated fungi (or bacteria) with numeric rankings (assessed as percentages or scores, depending on the system). As with any identification system, it is critical to have a comprehensive and well-curated database; this has been a notable limitation of historical fungal databases, especially those for dimorphic and filamentous fungi. Depending on relatedness of the test organism to the top match (and allowing for the next best matches), the organism is then identified at the group, complex, genus, species or subspecies-level. Usually, organisms are either appropriately identified or yield a low match, indicating that identification has not been attained; the latter suggests that the species being tested is not in the database, or that there is heterogeneity in individual species or genera, but may occur due to an insufficient amount of biomass being tested or poor technical preparation (in which case repeat testing, or testing after incubation for further growth, may be helpful). A Clinical and Laboratory Standards Institute guideline on MALDI-ToF MS was published in 2017 [5].

In the past, fungal identification has been a perplexing, multi-step process, tailored by organism-type. Clinical microbiology students were pedantically educated to interpret colony and microscopic morphology of fungi on solid media as a preface to choosing appropriate further testing, such as biochemical tests or sequencing. With MALDI-ToF MS, cultured yeasts may be correctly identified in minutes without a priori knowledge of organism-type; since it doesn’t matter whether a bacterium or yeast is being tested, the decision-making procedure characteristically surrounding differentiation of bacteria or yeasts growing on solid media prior to selecting further testing is obviated. Filamentous fungi can also be identified, though usually their processing prior to MALDI-ToF MS analysis typically takes longer than yeasts. MALDI-ToF MS is enabling implementation of total laboratory automation in clinical microbiology laboratories, allowing automated specimen processing, plating, incubation, plate reading using digital imaging, and spotting to MALDI-ToF MS plates. Early growth detection by digital imaging, paired with MALDI-ToF MS may result in earlier detection of fungi than conventional techniques [6]. MALDI-ToF MS is also changing the educational needs of clinical microbiology laboratory management staff, medical technologists, as well as medical students, fellows and residents. For the curriculum of those who won’t practice laboratory medicine, conventional biochemical-based identification is being deemphasized.

MALDI-ToF MS instruments used in clinical microbiology laboratories are typically specific for clinical microbiology applications, though other testing may be performed on them and alternative instruments may be used for clinical microbiology purposes. For purposes of efficiency and biosafety, however, such instruments are typically located in (or near) clinical microbiology laboratories themselves, rather than in a centralized mass spectrometry core facility. Commercial MALDI-ToF MS systems for clinical microbiology laboratories are available from bioMérieux, Inc. (Durham, NC, USA) and Bruker Daltonics, Inc. (Billerica, MA, USA). (Other systems, such as Andromas (Paris, France), Clin-TOF (China) Quan TOF (China), Autof ms 1000 (China), and Microtyper MS (China) will not be discussed). In 2010, bioMérieux acquired a microbial database called Spectral Archiving and Microbial Identification System (SARAMIS) marketed by AnagnosTec (Zossen, Germany) and used with Shimadzu’s AXIMA Assurance mass spectrometer (Shimadzu, Columbia, MD, USA), and transformed the label to VITEK MS research use only (RUO); bioMérieux then established a new database, software, and algorithms called VITEK MS IVD. bioMérieux’s FDA-approved/cleared platform, available since 2013, is named Vitek MS. A RUO version, VITEK MS Plus is available, incorporating the VITEK MS and SARAMIS databases. Bruker began developing a system for identification of cultured microorganisms circa 2005, the so-called Biotyper system, obtaining FDA-approval/clearance shortly after bioMérieux in 2013, with a system referred to as the MALDI Biotyper CA System. Like bioMérieux, Bruker offers a more extensive RUO database. Bruker also has a specific RUO Filamentous Fungi Library. Bruker’s mass spectrometer used for clinical microbiology testing is a desktop system, whereas bioMérieux’s is a larger instrument that sits on the floor.

Yeasts and filamentous fungi claimed by the FDA cleared/approved versions of at least one commercial MALDI-ToF MS system are shown in Table 1 and Table 2, respectively. Both companies’ systems claim an extensive portfolio of yeasts commonly encountered in clinical practice, though there are some nomenclature differences and inclusion differences. In some cases, one system may use the teleomorph with the other using the anamorph name; for example, *Cyberlindnera jadinii* is officially claimed by the MALDI Biotyper CA system, whereas *Candida utilis* is officially claimed by the Vitek MS system. The MALDI Biotyper CA system claims *Trichosporon mucoides* group (which per the company’s package insert includes *Trichosporon mucoides* and *Trichosporon dermatis*), whereas the Vitek MS system claims *T. mucoides*. Reporting of *Cryptococcus neoformans* and *Cryptococcus gattii* varies between the two systems (Table 1). Species uniquely claimed by the MALDI Biotyper CA system include *Candida boidinii*, *Candida duobushaemulonii*, *Candida metapsilosis*, *Candida orthopsilosis*, *Candida pararugosa*, *Candida valida*, and *Geotrichum candidum*, with *Candida rugosa* being uniquely claimed by the Vitek MS system. The Bruker and bioMérieux systems are different not just in databases, but also in database matching and relatedness reporting strategies. In most comparative studies, performance of the two has been similar, though not identical, assuming that the specific species being studied are represented in both databases [9,10,11]. Since there have been iterative and rapid growths and curations in both companies’ databases over time, in reviewing the published literature, it is important to note not just the company whose system was studied, but also the specimen preparation method and the library version applied, alongside the cutoffs used for identification at the species-, genus-, group- or complex-level. The organism testing sets studied (i.e., supplemented with unusual organisms or not), and reference (i.e., comparator) identification procedures should also be considered. With both systems, users have the option to develop their own database(s), which can enhance performance; this, however, makes generalization to other users challenging. In addition, user-developed databases must be validated to meet regulatory requirements for clinical use. User-developed databases can be used in conjunction with commercial databases; alternatively or additionally, multiple databases from the same company can be used together. Success rates may be compromised if spectra in a particular library were not created from isolates prepared in the same way as they are being tested (e.g., on-plate formic acid preparation versus off-plate protein extraction) [12].

MALDI-ToF MS turnaround time is five or fewer minutes per isolate for direct target plate methods; the turnaround time is longer with off-plate protein extraction. Compared to standard methods, yeast and bacterial identification is achieved an average of 1.45 days faster [14], and since only a slight amount of organism is required, testing can be completed on small amounts of growth on primary culture plates without subculture. MALDI-ToF MS has a low reagent cost [14], being less expensive than biochemical- or sequencing-based identification. One study showed that a projected 87% of bacterial and yeast isolates may be identified on the first day using MALDI-ToF MS (versus 9% historically) [14]. Using MALDI-ToF MS, DNA sequencing expenses can be avoided, waste disposal reduced [15,16], and quality control and technologist labor/training for retired tests/replaced tests avoided.

## 2. Yeasts, with a Focus on *Candida* and *Cryptococcus* Species

MALDI-ToF MS has rapidly become a standard method for yeast identification, out-performing some historical phenotypic systems, and differentiating *Candida albicans* from *Candida dubliniensis*; *C. pararugosa* from *Candida rugosa*; *Candida krusei*, *Candida norvegensis*, and *Candida inconspicua* from one another; *C. orthopsilosis*, *C. metapsilosis* and *C. parapsilosis* from one another [17]; and *C. gattii* from *C. neoformans*, dependent on spectral database representation [18,19]. MALDI-ToF MS may outdo other identification systems for esoteric species, such as *C. famata* and *C. auris*, for example [12,17]. Although older studies used off-plate extraction for yeasts, on-plate extraction with formic acid is now favored for its simplicity; on-plate formic acid preparation yields higher identification rates than does direct transfer alone [20,21].

Dhiman et al. evaluated the Bruker system for identification of 138 common and 103 unusual yeast isolates, reporting 96% and 85% accurate species-level identification, respectively [22]. Westblade et al. assessed the Vitek MS v2.0 system for identification of 852 yeast isolates, including *Candida* species, *C. neoformans*, and other clinically relevant yeasts, using on-plate formic acid preparation, in a multicenter study, reporting 97% and 86% identification to the genus- and species-level, respectively [20]. Won et al. assessed the accuracy of yeast bloodstream isolate identification using the Vitek MS system; correct identification, misidentification and no identification were achieved in 96%, 1% and 3% of cases, respectively [23]. Mancini et al. compared the Bruker and Vitek MS systems for identification of yeasts; correct species-level identifications were comparable using the commercial databases (90% and 84%, respectively), with 100% identified using the Bruker system and a user-developed database [24]. More misidentifications were reported with the Vitek MS system compared to the Bruker system. Rosenvinge et al. studied the Bruker system with 200 yeast isolates, reporting 88% species-level identification (species cutoff of ≥1.700) using on-plate formic acid testing [25]. Lacroix et al. demonstrated that the Bruker system with protein extraction and using the manufacturer’s species-level cutoff identified 97% of 1383 regularly isolated *Candida* isolates [26]. Pence et al. compared the VITEK MS (IVD Knowledgebase v.2.0) and Biotyper (software v3.1) for identification of 117 yeast isolates, showing correct identification of 95% and 83% of isolates, respectively, using on-plate formic acid testing [27]. Jamal et al. evaluated the Bruker and VITEK MS systems for identification of 188 clinically significant yeast isolates [28], reporting accurate identification of 93% of isolates with both. Three isolates were not identified by VITEK MS, while nine *C. orthopsilosis* were incorrectly identified as *C. parapsilosis*, which was not unexpected since *C. orthopsilosis* was not included in the database studied. Eleven isolates were not identified or misidentified by the Bruker system and although another 14 were identified correctly, their score was <1.700. Hamprecht et al. compared the VITEK MS (V2.0 knowledge base) and the Biotyper (v3.0 software, v3.0.10.0 database, using a species-level cutoff ≥2.000) systems for identification of 210 yeasts using on-plate formic acid testing, showing identification of 96% and 91%, respectively [29]. De Carolis et al. made an in-house database using spectra from 156 reference and clinical yeast isolates generated with a sample preparation procedure using suspension of a colony in 10% formic acid, and using 1 µL of the lysate for analysis [30]. Using their library and processing method, and the Bruker system (software v3.0) with a species-level cutoff of ≥2.000, they identified 96% of 4232 routinely isolated yeasts. Fatania et al. evaluated the Bruker system with 200 clinically significant yeasts, representing 19 species and five genera, showing agreement between MALDI-ToF MS and conventional methods for 91% [31]. Wang et al. evaluated 2683 yeast isolates comprising 41 species from the National China Hospital Invasive Fungal Surveillance Net program, reporting that the Bruker Biotyper MS system exhibited greater accuracy than the Vitek MS system for all isolates (99% and 95%, respectively) and for *Candida* and related species (99% and 96%, respectively) [32]. Fraser et al. evaluated MALDI-ToF MS using the Bruker system for identification of 6343 clinical isolates of yeasts representing 71 species using a user-developed simplified rapid extraction method, reporting correct identification of 94% of isolates, with a further 6% identified after full extraction [33]. Lee et al. compared the Bruker and VITEK MS systems for identification of 309 clinical isolates of four common *Candida* species, *C. neoformans*, as well as 37 uncommon yeast species, using on-plate formic acid preparation [34]. If “no identification” was obtained, isolates were retested using on-plate formic acid preparation and, for the Bruker system, tube-based extraction. Both systems accurately identified all 158 isolates of the common *Candida* species with initial analysis. The Bruker system correctly identified 9%, 30%, and 100% of 23 *C. neoformans* isolates after initial on-plate formic acid preparation, repeat on-plate formic acid preparation, and tube-based extraction, respectively; VITEK MS identified all *C. neoformans* isolates after initial on-plate formic acid preparation. Both systems had comparable identification rates for 37 uncommon yeast species following initial on-plate formic acid preparation (Bruker, 74%; VITEK MS, 73%) and repeat on-plate formic acid preparation (Bruker, 82%; VITEK MS, 73%). Marucco et al. compared identification of *Candida* species obtained by BD Phoenix (Becton Dickinson, Franklin Lakes, NJ, USA) and the Bruker system using 192 isolates from the strain collection of the Mycology Network of the Autonomous City of Buenos Aires, Argentina, reporting an observed concordance of 95%, with 5% of isolates not correctly identified by the BD Phoenix system [35]. Wilson et al. reported results of a multicenter assessment of the Bruker MALDI Biotyper CA system for identification of clinically significant bacteria and yeasts, including 815 yeast isolates evaluated using three processing methods [36]. The percentage identified and the percentage identified with a high level of confidence were 98% and 88%, respectively, with the extended direct transfer method being superior to the direct transfer method (74% and 49% success, respectively) [36]. Turhan et al. assessed the Bruker system with 117 yeasts, including 115 candidemia-associated *Candida* species, reporting 98% and 87% identification to the genus- and species-level, respectively [37]. Porte et al. compared the two commercial MALDI-ToF MS systems in a routine laboratory in Chile, in a study that included 47 yeasts; the bioMérieux system yielded higher rates of yeast identification to species-level than did the Bruker system (46 and 37 respectively) [38].

### 2.1. *Malassezia* Species

*Malassezia furfur* and *Malassezia pachydermatis* are included in both FDA-approved/cleared databases (Table 1). Denis et al. developed and evaluated a MALDI-ToF MS database for identifying *Malassezia* species using the Bruker system [39]. Forty-five isolates of *M. furfur*, *Malassezia slooffiae*, *Malassezia sympodialis*, *M. pachydermatis*, *Malassezia restricta* and *Malassezia globosa* were used to create a database, with 40 different isolates used to test the database; all isolates were identified with scores of >2.000.

### 2.2. *Trichosporon* Species

*Trichosporon inkin* and *Trichosporon asahii* are included in both FDA-approved/cleared databases, with *T. mucoides* additionally claimed by the Vitek MS database and *T. mucoides* group claimed by the MALDI Biotyper CA system (Table 1). de Almeida et al. subjected 16 *Trichosporon* species isolates to MALDI-ToF MS using the Bruker system, evaluating several extraction methods [40]. Overall, incubation for 30 min with 70% formic acid yielded spectra with the highest scores; among the six libraries studied, a library made of 18 strains plus seven clinical isolates yielded the best results, correctly identifying 99% of 68 clinical isolates.

## 3. Filamentous Fungi

Filamentous fungi demonstrate variable phenotypes as a result of which protein spectra may vary; heterogeneity can be affected by growth conditions and the zone of fungal mycelium examined. Nevertheless, filamentous fungi can be identified using MALDI-ToF MS [41]. The FDA-approved/cleared Vitek MS system claims 47 filamentous fungi, either species or complexes, including dimorphic pathogens, alongside dermatophytes. As mentioned above, Bruker has an RUO Filamentous Fungi Library. Sample preparation has varied from study-to-study, with sample preparation for molds recommended by companies having changed over time [41]; the FDA-approved/cleared Vitek MS system uses off-plate protein extraction.

McMullen et al. evaluated the Vitek MS using the Vitek MS Knowledge Base, v3.0 for identification of 319 mold isolates, representative of 43 genera, reporting 67% correct identification; when a modified SARAMIS database was used to supplement the v3.0 Knowledge Base, 77% were identified [42]. Rychert et al. reported correct species-level identification of 301/324 clinical isolates of various *Aspergillus* species tests as part of an FDA trial of the Vitek MS v3.0 system; species evaluated included *Aspergillus brasiliensis, Aspergillus calidoustus, Aspergillus flavus*/*oryzae, Aspergillus fumigatus, Aspergillus lentulus, Aspergillus nidulans*, *Aspergillus niger* complex, *Aspergillus sydowii, Aspergillus terreus* complex, and *Aspergillus versicolor* [43]. Rychert et al. also reported correct species identification of 205/325 clinical isolates of dematiaceous fungi in the same study, including *Alternaria alternata, Curvularia hawaiiensis, Curvularia spicifera*, *Exserohilum rostratum*, *Exophiala dermatitidis*, *Exophiala xenobiotica*, *Scedosporium boydii*, *Scedosporium apiospermum*, *Scedosporium prolificans* and *Cladophialophora bantiana* [43]. Finally, Rychert et al. reported correct species-level identification of 298/315 clinical isolates of “other potential pathogens”, including *Fusarium oxysporum* complex, *Fusarium proliferatum*, *Fusarium solani* complex, *Paecilomyces variotii, Penicillium chrysogenum*, *Rasamsonia argillacea*, *Acremonium sclerotigenum, Lecythophora hoffmannii*, *Sarocladium kiliense* and *Purpureocillium lilacinum* [43].

De Carolis et al. established their own library of *Fusarium* species, *Aspergillus* species, and Mucorales using the Biotyper system and identified 97% of 94 isolates to the species-level [44]. Gautier et al. used an in-house database to assess the level to which MALDI-ToF MS performed using the Bruker platform enhanced identification; implementation of MALDI-ToF MS resulted in marked enhancement in mold identification at the species-level (to 98%) [45]. Lau et al. used a special extraction technique with a user-developed library representing 294 isolates of 76 genera and 152 species and the Bruker system, to test 421 mold isolates, achieving correct species- and genus-level identifications of 89% and 93% of isolates, respectively [46]. Zvezdanova et al. recently assessed the Bruker system with the Filamentous Fungi Library 1.0 for clinical mold identification using direct target plate testing and simplified processing consisting of mechanical lysis of molds preparatory to protein extraction [47]. They reported accurate species-level identification of 25/34 *Fusarium* species and all 10 *Mucor circinelloides* isolates tested. In addition, 1/21 *Pseudallescheria*/*Scedosporium and* 7/34 *Fusarium* species isolates were correctly identified to the genus level. The remaining 60 isolates were not identified using the commercial database. They then constructed an in-house database with 63 isolates, which allowed identification of 91% and 100% identification to the species- and genus-levels, respectively.

Normand et al. reported decision criteria for MALDI-ToF MS identification of molds and dermatophytes using the Bruker system [48]. They employed user-developed and Bruker databases as well as 422 isolates of 126 species to evaluate a number of thresholds and one to four spots. They found optimal results with a decision algorithm in which only the uppermost score of four spots was applied, with a 1.700 score threshold. Testing the complete panel enabled identification of 87% and 35% of isolates with the user-developed and Bruker databases, respectively. Applying the same rules to isolates with species represented by at least three strains in the database allowed identification of 92% and 47% of isolates with the user-developed and Bruker databases, respectively. Huang et al. described their findings using the Bruker system and 374 clinical filamentous fungal isolates with correct species and genus identification realized in 99% and 100% of isolates, respectively [49]. Riat et al. used the Bruker Filamentous Fungi Library 1.0, reporting that an identification score of >1.700 was obtained for 92% of 48 mold isolates studied [50]. Using the Bruker system and a user-developed database, Masih et al. identified 95% of *Aspergillus* species [51]. Park et al. evaluated the Bruker’s Filamentous Fungi Library 1.0 with 345 clinical *Aspergillus* isolates; compared with findings of internal transcribed spacer (ITS) sequencing, rates of accurate identification at the species-complex level were 95% and 99%, with cutoff values of 2.000 and 1.700, respectively [52]. Compared with β-tubulin gene sequencing, rates of accurate identification to the species-level were 96% (cutoff 2.000) and 100% (cutoff 1.700) for 303 *Aspergillus* isolates of five common species, but only 5% (cutoff 1.700) and 0% (cutoff 2.000) for 42 *Aspergillus* isolates of six rare species. Schulthess et al. evaluated Bruker’s Filamentous Fungi Library 1.0, first studying 83 phenotypically- and molecularly-characterized, non-dermatophyte, non-dematiaceous molds from a clinical isolate collection [53]. Using manufacturer-recommended interpretative criteria, genus and species identification frequencies were 78% and 54%, respectively. Decreasing the species cutoff to 1.700 increased species identification to 71%, without impacting misidentification. In a follow-on prospective study, 200 successive clinical mold isolates were assessed; genus and species identification rates were 84% and 79%, respectively, with a species cutoff of 1.700. Sleiman et al. developed a database for identification of *Aspergillus*, *Fusarium* and *Scedosporium* species [54]. Using 117 isolates, species-level identification was enhanced when the user-developed database was used in conjunction with the Bruker Filamentous Fungi Library compared with the Bruker database alone (*Aspergillus* species, 93% versus 69%; *Fusarium* species, 84% versus 42%; and *Scedosporium* species, 94% versus 18%, respectively). Becker et al. employed a user-developed library and the Bruker system to evaluate 390 clinical isolates, reporting correct identification of 86% of isolates to the species-level using a cutoff of 1.700 [55]. Vidal-Acuña et al. created their own library using 42 clinical *Aspergillus* isolates and 11 strains, cultured in liquid medium, including 23 different species [56]. One hundred and ninety isolates cultured on solid media (179 clinical isolates identified by sequencing and the 11 strains) were studied, with species- and genus-level identifications of 87 and 100%, respectively. They then prospectively challenged their library with 200 *Aspergillus* clinical isolates grown on solid media; species identification was obtained in 96%. Stein et al. evaluated the Bruker system with clinical isolates and reference strains of molds using the Bruker mold, National Institutes of Health, and Mass Spectrometry Identification (MSI) online libraries, comparing results to morphological and molecular identification methods [57]. All libraries studied showed better accuracy in genus identification (≥95%) compared to conventional methods (86%), with 73% of isolates identified to the species-level. The MSI library showed the highest rate of species-level identification (72%) compared to National Institutes of Health (20%) and Bruker (14%) libraries. More than 20% of molds were unidentified to the species-level by all libraries studied, a finding attributed to library limitations and/or poor spectra. Triest et al. evaluated the Bruker system with a user-developed database for identification of 289 *Fusarium* isolates encompassing 40 species from the Belgian Coordinated Collections of Microorganisms/Institute of Hygiene and Epidemiology Mycology culture collection, observing no incorrect species complex identifications [58]. 83% of identifications were accurate to the species-level.

Rychert et al. reported correct species-level identification of clinical isolates of 24/30 *Mucor racemosus* complex, 22/28 *Rhizopus arrhizus* complex, 26/29 *Rhizopus microsporus* complex and 29/31 *Lichtheimia corymbifera*, as part of an FDA trial of the Vitek MS v3.0 system [43]. Dolatabadi et al. utilized the Bruker system with a user-developed database for identification of *R. arrhizus* and its varieties, *delemar* and *arrhizus*, as well as *R. microspores* [59]. Chen et al. assessed the Bruker system with 50 clinically encountered mold isolates, including *Talaromyces marneffei*, *Rhizopus* species, *Paecilomyces* species, *Fusarium solani*, and *Pseudallescheria boydii* [60]. The correct identification rate of *T. marneffei* (score ≥2.000) was 86% based on their user-developed library. Although all seven *P. variotii* isolates, two of the four *P. lilacinus*, four of the six *F. solani*, and two of the three isolates of *Rhizopus* species, and the *P. boydii* isolate had concordant identifications between MALDI-ToF MS and sequencing analysis, scores were all <1.700 [60]. Shao et al. studied 111 isolates of Mucorales belonging to six genera from the Research Center for Medical Mycology of Peking University, initially using the Bruker Filamentous Fungi library (v1.0), showing 50% and 67% identification to species- and genus-levels, respectively [61]. They then created an in-house library, the Beijing Medical University database, using [11] strains of *Mucor hiemalis*, *Mucor racemosus*, *Mucor irregularis*, *Cunninghamella phaeospora*, *Cunninghamella bertholletiae*, and *Cunninghamella echinulate*. Using the Beijing Medical University and Bruker databases together, all 111 isolates were identified, 81% and 100% to the species- and genus-levels, respectively. 

Singh et al. analyzed 72 melanized clinical fungal isolates from patients in 19 Indian medical centers using the Bruker system and a user-developed database, reporting 100% identification [62]. Paul et al. created an in-house database of 59 melanized fungi using a modified protein extraction protocol, and tested 117 clinical isolates using the database [63]. Whereas using the Bruker database only 29 (25%) molds were identified, all were accurately identified accurately by supplementing the Bruker database with the in-house library.

### Dermatophytes

With appropriate databases, dermatophytes may be identified using MALDI-ToF MS [64,65,66]. *Microsporum audouinii, Microsporum canis, Microsporum gypseum, Epidermophyton floccosum, Trichophyton rubrum, Trichophyton interdigitale, Trichophyton tonsurans, Trichophyton verrucosum*, and *Trichophyton violaceum* are included in the FDA-approved/cleared Vitek MS system, with no dermatophytes in the FDA-approved/cleared Bruker system. Rychert et al. reported correct species-level identification of clinical isolates of 30/33 *M. audouinii*, 30/31 *M. canis*, 32/35 *M. gypseum*, 30/31 *E. floccosum*, 31/31 *T. rubrum*, 29/30 *T. interdigitale*, 30/33 *T. tonsurans*, 18/31 *T. verrucosum*, and 13/34 *T. violaceum*, as part of an FDA trial of the Vitek MS v3.0 system [43].

Packeu et al. evaluated the Bruker system with a user-developed library for the identification of 176 clinical dermatophyte isolates [67]. MALDI-ToF MS yielded accurate identifications of 97 and 90% of isolates with lowered scores and application of the user-supplemented database, respectively, versus 52% and 14% correct identifications with the unmodified library and recommended scores at the genus- and species-levels, respectively. Calderaro et al. determined the ability of a user-developed database with the Bruker system to identify 64 clinical isolates; all were correctly identified (score of >2.000 for 47 isolates, and 1.700 to 2.000 for the other 17 isolates) [68]. An on-plate procedure after 3 days of incubation produced 40% accurate identification; prolonging incubation time and using an extraction procedure both yielded 100% accurate identification. Karabicak et al. evaluated the Bruker system using a user-developed database with 126 dermatophytes, including 115 clinical isolates and [9] strains; using a combination of the user-developed database and lowered cutoff scores, genus and species identifications were achieved for 97% and 90% of the isolates [69]. L’Ollivier et al. appraised ten studies published between 2008 and 2015 showing accuracy of MALDI-ToF MS-based identification of dermatophytes to vary between 14 and 100% [70]; they ascribed inconsistencies, in part, to processing variability. Use of a tube-based extraction step and a manufacturer database augmented with user-developed spectra were helpful for accurate species identification. Da Cunha et al. assessed whether the direct transfer method can be used with dermatophytes [71]. They built their own library using the Bruker system and evaluated its performance with a panel of mass spectra produced with molecularly-identified isolates and, compared MALDI-ToF MS to morphology-based identification. Although dermatophyte identification using the Bruker library was poor, their database yielded 97% concordance between ITS sequencing and MALDI-ToF MS with 276 isolates. The direct transfer method using unpolished target plates permitted the correct identification of 85% of the clinical dermatophyte isolates.

## 4. Dimorphic Fungi

The Vitek MS database includes *Blastomyces dermatitidis*, *Coccidioides immitis*/*posadasii, Histoplasma capsulatum*, and *Sporothrix schenckii* complex (Table 2); Rychert et al. evaluated 40, 38, 32 and 31 of these, respectively, as part of an FDA trial of the Vitek MS v3.0 system, reporting 100% identification [43]. Lau et al. assessed the Bruker system for identification of 39 isolates of *T. marneffei* [72]. Using the Filamentous Fungi Library 1.0, MALDI-ToF MS did not identify the isolates; when the database was expanded by including spectra from 21 *T. marneffei* isolates, all isolates in the mold or yeast phase were identified to the species-level. De Almeida et al. showed that the Bruker system with a user-developed database, could identify *Paracoccidioides brasiliensis* and *Paracoccidioides lutzii* [73]. Valero et al. established their own *H. capsulatum* Bruker database using six strains [74]. Then, 30 *H. capsulatum* isolates from the Collection of the Spanish National Centre for Microbiology were studied and correctly identified, 87% with scores above 1.700. The created database was able to identify both growth phases of the fungus, with the most reliable results for the mycelial phase.

## 5. Limitations

MALDI-ToF MS has limitations. Unlike publicly available sequence databases, such as GenBank, commercial MALDI-ToF MS databases are typically exclusive to companies. Although low identification rates for some organisms may be enhanced by user addition of mass spectral entries of underrepresented species or strains (to cover intraspecies variability), or even re-addition of reference strain spectra to the library, especially those created using parallel growth conditions and preparation methods, doing so may be beyond the know-how of some laboratories. Because of low scores/percentages, repeat testing of isolates may be required [14]. Growth on some media may yield low scores/percentages [75], and small or mucoid colonies may fail. Using experimental capsule size manipulation, it was demonstrated that capsule size of *C. neoformans* and *C. gattii* can compromise identification by the Bruker system [76]. Refined interpretive criteria may be needed to discriminate closely related species and distinguish them from the next best taxon match. For some species of fungi, genus- or species-specific (including lowered) cutoffs may be needed. Mistakes that may occur include testing mixed colonies, spreading amongst spots, spotting into incorrect target plate positions, not properly cleaning re-usable target plates, and wrongly entering results into laboratory information systems. There is a learning curve to depositing ideal biomass onto target plates [77]. Although results are normally reproducible, sources of variability include the technologist, mass spectrometer and especially laser age, matrix and solvent make-up, biological variability, and culture conditions [48]. Instrument (e.g., laser) and software failure may happen. As a result of the simplicity of MALDI-ToF MS, technologists may lose or never develop fine-tuned abilities to visually identify fungi, macroscopically and microscopically.

## 6. Conclusions

In summary, MALDI-ToF MS has become a routine method for the identification of yeasts and is also being applied to filamentous and dimorphic fungi. Although databases are slowly becoming more complete with regards to clinically-relevant fungi, due to evolving nomenclature and constant description of new species/genera, systematic and continuing library updates will be needed to deliver quality fungal identification into the anticipatable future.

This manuscript has its limitation as the appreciation of MALDI-ToF MS to fungi is rapidly evolving such that some of the cited studies, even if recently published, may rapidly be antiquated. 

This paper is based in part on [4,7,8,13,78].

## Figures and Tables

**Table 1:** Reportable yeasts for the FDA-approved/cleared Vitek MS and MALDI Biotyper CA systems as of October 2018 [13]. Those entries marked with “^V^” are FDA-approved/cleared for the Vitek MS system only and those marked with “^B^” are FDA-approved/cleared for the MALDI Biotyper CA system only. Those with marked with neither a “^V^” nor a “^B^” are FDA-approved/cleared on both systems.

*Candida albicans*	*Candida krusei*	*Candida tropicalis*	*Kodamaea/Pichia ohmeri* ***
*Candida boidinii* ^B^	*Candida lambica*	*Candida utilis/Cyberlindnera jadinii* *	*Malassezia furfur*
*Candida dubliniensis*	*Candida lipolytica*	*Candida valida* ^B^	*Malassezia pachydermatis*
*Candida duobushaemulonii* ^B^	*Candida lusitaniae*	*Candida zeylanoides*	*Rhodotorula mucilaginosa*
*Candida famata*	*Candida metapsilosis* ^B^	*Cryptococcus gattii* ^B^	*Saccharomyces cerevisiae*
*Candida glabrata*	*Candida norvegensis*	*Cryptococcus neoformans* ^V^	*Trichosporon asahii*
*Candida guilliermondii*	*Candida orthopsilosis* ^B^	*Cryptococcus neoformans *var* grubii* ^B^	*Trichosporon inkin*
*Candida haemulonii*	*Candida parapsilosis*	*Cryptococcus neoformans *var* neoformans* ^B^	*Trichosporon mucoides* ^V^
*Candida inconspicua*	*Candida pararugosa* ^B^	*Geotrichum candidum* ^B^	*Trichosporon mucoides* group ^B^
*Candida intermedia*	*Candida pelliculosa*	*Geotrichum capitatum/Saprochaete capitate* **	
*Candida kefyr*	*Candida rugosa* ^V^	*Kloeckera apiculata*	

* *Cyberlindnera jadinii* (teleomorph) is approved/cleared on the MALDI Biotyper CA system, whereas *Candida utilis* (anamorph) is approved/cleared on the Vitek MS system. ** Geotrichum capitatum is approved/cleared on the MALDI Biotyper CA system, whereas *Saprochaete capitate* is approved/cleared on the Vitek MS system **; *** *Kodamaea ohmeri* is approved/cleared on the Vitek MS system whereas *Pichia ohmeri* is approved/cleared on the MALDI Biotyper CA system.

**Table 2:** Reportable filamentous and dimorphic fungi for the FDA-approved/cleared Vitek MS system as of October 2018 [13].

*Acremonium sclerotigenum*	*Blastomyces dermatitidis*	*Histoplasma capsulatum*	*Rhizopus arrhizus* complex
*Alternaria alternata*	*Cladophialophora bantiana*	*Lecythophora hoffmannii*	*Rhizopus microsporus* complex
*Aspergillus brasiliensis*	*Coccidioides immitis/posadasii*	*Lichtheimia corymbifera*	*Sarocladium kiliense*
*Aspergillus calidoustus*	*Curvularia hawaiiensis*	*Microsporum audouinii*	*Scedosporium apiospermum*
*Aspergillus flavus/oryzae*	*Curvularia spicifera*	*Microsporum canis*	*Scedosporium prolificans*
*Aspergillus fumigatus*	*Epidermophyton floccosum*	*Microsporum gypseum*	*Sporothrix schenckii* complex
*Aspergillus lentulus*	*Exophiala dermatitidis*	*Mucor racemosus* complex	*Trichophyton interdigitale*
*Aspergillus nidulans*	*Exophiala xenobiotica*	*Paecilomyces variotii* complex	*Trichophyton rubrum*
*Aspergillus niger* complex	*Exserohilum rostratum*	*Penicillium chrysogenum*	*Trichophyton tonsurans*
*Aspergillus sydowii*	*Fusarium oxysporum* complex	*Pseudallescheria boydii*	*Trichophyton verrucosum*
*Aspergillus terreus* complex	*Fusarium proliferatum*	*Purpureocillium lilacinum*	*Trichophyton violaceum*
*Aspergillus versicolor*	*Fusarium solani* complex	*Rasamsonia argillacea* complex	
